# New Root Filling

**Published:** 1919-10

**Authors:** W. Clyde Davis


### NEW ROOT FILLING

W. CLYDE DAVIS, M.D., D.D.S.

This preparation has all the advantages found in former combinations and most of the faults eliminated. The material is prepared as follows:

Chloroform, 1½ drams; Rubber (pure gum), 4 grains; Rosin, 16 grains; Di-thymol Di-iodid, 4 grains. These should be combined and allowed to stand for twenty-four hours, then add one-half dram eucalyptol. By a careful study of the properties of each one of these ingredients I believe the material will meet with favor.

The chloroform is the main solvent. The pure rubber gum is that which is called raw rubber and can be had at any automobile tire repair shop. This amount of rubber assists in the formation of a protecting skin to prevent evaporation, which is one of the virtues of the Callahan varnish. It will be noted that the rosin in this preparation is much heavier than with the Callahan varnish. This is made possible by the presence of eucalyptol which prevents rapid evaporation of the chloroform. The eucalyptol also has the property of raising the moisture from the sides of the canal, provided by accident the canal is not entirely dry.

In the use of this preparation the finest point can be pushed entirely to place before the chloroform has sufficiently weakened the point to cause it to bend. Within a minute or two this point will have been sufficiently disintegrated to cause it to bend, so as to prevent the accident of pushing the same too far through the root canal.

The uses of the di-thymol di-iodid are apparent. This also assists the radiographer, for when a root canal has been filled with this preparation the roentgenogram will show when a root canal has been imperfectly filled, as the point will show white. The part which has been filled with this fluid only will show gray and that part of the canal which has not been filled with either will show dark. This is a most valued fact in the use of this preparation, as all root canals should be filled with gutta-percha, sealed with a sealing fluid. This preparation will be found most adhesive and can not be removed from a wet surface except by the use of a solvent.

All the materials are easily obtained by any dentist and are inexpensive. The percentage of sore teeth following the placing of a root filling is much less than with any other preparation we ever have used.

Before using, all canals should be flooded with eucalyptol, then excess absorbed out so that the walls are only damp with eucalyptol.—*Dental Summary*, for July, 1919.

